# Polycystic ovary syndrome dependency on mtDNA mutation; copy Number and its association with insulin resistance

**DOI:** 10.1186/s13104-019-4453-3

**Published:** 2019-07-24

**Authors:** Noor AlHuda Ali A. H. Saeed, Israa Hussein Hamzah, Samar Abdul Raheem Al-Gharrawi

**Affiliations:** 1 Branch of Zoology, Biology Department, College of Science, Mustansiriyah University, POX 10422, Baghdad, Iraq; 2 Dept. of Basic Science, College of Dentistry, Mustansiriyah University, POX 10422, Baghdad, Iraq

**Keywords:** MtDNA, PCOS, Copy number, Mutation, Diabetes, Maternal inheritance

## Abstract

**Objective:**

Study analyzes mutation in mtDNA (Mitochondrial DNA) among diabetic women with PCOS in non-diabetic diabetic women and compared with the healthy control. Women with known case of hyperandrogenism, ovulatory dysfunction and/or polycystic ovaries were selected and anthropometric and demographic variables were collected during their clinical visit. Biochemical estimation of glucose, FSH, LH, estradiol (E2), and insulin levels were analyzed. Mutational analysis of mt-tRNA genes of each individual was compared with the updated consensus Cambridge sequence. The mtDNA content was determined in triplicate using SYBR green PCR mastermix.

**Results:**

The clinical and biochemical characteristics of participants showed no statistical difference in age and/or FSH, PRL, E2, PRGE or fasting glucose value between patients of different groups. Women with PCOS-D had significantly higher LH, LH/FSH, TT and fasting insulin levels and HOMA-IR with respect to the control group. Ten different type of mutation were seen in POCS group. Most of these mutations were confined to evolutionarily conserved region. The mtDNA copy numbers were considerably lower PCOS group irrespective of diabetic status. To conclude, the current study inferred that the mutations occur in the mitochondrial genome, mt-tRNA in specific, are the important causal factor in PCOS.

## Introduction

Polycystic ovary syndrome (PCOS) affects women up to 5–10% during their reproductive age and is an endocrine-dependent multifactorial symptom such as ovulatory dysfunction, hyperandrogenism, anovulation, and polycystic ovarian morphology [1]. The presence of a number of cysts in the ovaries is the predominant characteristic of this syndrome which results in the condition termed as ‘hyperandrogenism’ and ovulatory dysfunction. There is an association between insulin resistance and PCOS in about 40% of the women which results in hormonal disturbance [2]. This hormonal disturbance is a critical factor associated with the syndrome. Various studies proved the presence of PCOS in women who are diagnosed with T2DM (Type 2 Diabetes Mellitus) though this is not clearly explored area in terms of molecular biology [3, 4].

Sometimes hyperglycemia may induce the excess production of ROS (reactive oxygen species) which eventually results in the malfunction of the mitochondria [5]. Cells and their cellular organs i.e. mitochondria play a vital role in the production of ATP required in a number of cellular processes, for instance glucose metabolism. T2DM has an association with maternal mitochondrial inheritance in addition to PCOS [6]. During glucose metabolism, some researchers suggested that the production of insulin might significantly vary as per the functioning of mitochondria [7–9]. Two genes such as mitochondrial oxidative phosphorylation genes and mitochondrial tRNA genes act as the dependent factor for glucose metabolism. If there is any mutation occurs in these genes, it eventually impacts the maternally-inherited diabetes according to Lee et al. [10] and Lee et al. [11] According to Liu et al. [9], mitochondria can be mentioned as a complete cell entity within a cell since it has indigenous DNA [10, 11]. The mitochondrial activities can be measured by the cellular activities of mitochondria DNA.

It is a well-accepted fact that any changes brought in the mitochondrial DNA genes results in its altered functions in association with peripheral IR and glucose intolerance [12, 13]. In order to understand this biological scenario, the current study is conducted to analyze the frequency of mutation in mtDNA among diabetic women with PCOS and compared it with non-diabetic women with PCOS and diabetic non PCOS women. The results would enable to evaluate the association of the mtDNA mutation with PCOS in diabetic and non diabetes women in this ethnic population.

## Main text

### Methods

In a prospective case controlled study conducted between January 2016 and December 2017, women’s of reproductive age with known case of PCOS as indicated by the 2003 Rotterdam Criteria (1) according to which women’s having two of three conditions hyperandrogenism, ovulatory dysfunction and/or polycystic ovaries were selected. Womens suffering with some other endocrine (thyroid disorders), hormonal imbalance, adrenal hyperplasias, Cushing’s syndrome were excluded from this study. Participants were enrolled in three group, Group I had 32 diabetic women’s with PCOS syndrome, Group II, includes 38 non diabetic PCOS women, Group III includes 34 Diabetic women but non-PCOS. Additionally, 25 age and Body Mass Index (BMI) matched healthy control participants were recruited.

Each individual participant’s anthropometric and demographic variables such as age, height, waist by hip ratio (WHR) weight, body Mass Index (BMI), and ethnicity, education status, occupation were collected during their clinical visit. Clinical signs of hyperandrogenism were assayed based on the presence of malepattern baldness, hirsuitism, oily face, acne, and body hair. This was further assayed and matched with results of ultra sound scan for polycystic ovaries and their volume of ovarian fluid and number follicles, endometrial thickness was screened in PCOS patients alone. Family history of diabetic and ovulatory dysfunction was obtained from each participants of study.

#### Laboratory assessment

On the first day of menstruation, early morning blood samples were collected from each participant after an overnight fast. Biochemical estimation of glucose, follicle stimulating hormone (FSH), Luteinizing hormone (LH), estradiol (E2), and insulin levels were analyzed by the fully automated chemiluminiscence techniques (Roche, Indianapolis). Post breakfast glucose levels were measured after 2 h of food intake using enzymatic techniques and autoanalyzer (Bayer Diagnostic, Tarrytown). The homeostasis model assessment (HOMA) index was calculated to obtain IR using formula as given in earlier literature.

The total DNA was isolated using automated DNA extraction machine (Qiamp DNA, USA) following the manufactures instruction. The polymerase chain reaction (PCR) was run to magnify the mt-tRNA genes (MT-TL1 mitochondrially encoded tRNA leucine 1 (UUA/G); Gene ID: 4567) of every participants using primers as given in published literature following their protocol (9). Each amplified product was purified and sequenced in an ABI automated DNA sequence using a Big Dye Terminator Cycle sequencing reaction kit. The resultant sequence data were compared with the updated consensus Cambridge sequence (GenBank accession number: NC_012920) [16, 17], using the Seqscape software v2.7.

The mtDNA content was determined in triplicate in using SYBR green PCR mastermix in QPCR (Applied Biosystems, Foster City) targeting the mitochondrial *ND1* gene and normalized with using endogenous control i.e. the nuclear human β-globins gene and [14]. The specific primers and probes used to amplify the nuclear β-globins and mitochondrial *ND1* genes were as follows: nuclear β-globin gene; forward, 5′-CTGGGCATGTGGAGACAGAGAAGACT; reverse, 5′-AGGCCATCACTAAAGGCACCGAGC, probe 5′-FAM-CCCTTAGGCTGCTGGTGGTCTACCCTTTAMRA. Mitochondrial *ND1* gene; forward, 5′-GACGCCATAAAACTCTTCACCAA, reverse, 5′-AGGTTGAGGTTGACCAGGGG, probe 5′-FAMCCATCACCCTCTACATCACCGCCC-TAMRA [15]. A melting curve was performed to ensure the purity of target gene amplification through specific melting temperatures using the Dissociation Curve Software. Ct value differences (delta Ct) were referred as absolute changes in mtDNA copy number relative to β-actin which was calculated as follows: relative copy number (Rc) = 2^ΔCt^, where ΔCt = Ct_β-actin_ − Ct_ND1_.

### Statistical analysis

Fisher exact “t” test was used to compare the frequencies of mutations between groups. Student *t* test was used to test the level of significance in the fold change. P-value < 0.05 was considered statistically significant.

### Results

The patients’ clinical characteristic was presented in Table 1. The group was closely similar for age, BMI and age varied between 24 and 25 years. The clinical and biochemical characteristics of participants enrolled in this study included showed no statistical difference in age and/or FSH, PRL, E2, PRGE or fasting glucose value between patients of different groups. The significant difference was seen in LH, LH/FSH, TT, insulin and HOMA-IR value among the groups. Women with PCOS-D had significantly higher LH, LH/FSH, TT and fasting insulin levels and HOMA-IR with respect to the control group and non PCOD-D group indicating their possible role in PCOS. The values of PCOS-ND were comparable to PCOS-D group further supports that the factors tested were mostly related to PCOS if they were significantly varying.

**Table 1:** Clinical and metabolic features of PCOS-IR patients and control subjects Characteristics

P	PCOS-D (n = 32)		PCOS-ND (n = 38)		NPCOS-D (n = 34)		HC (n = 25)	
	Mean	SD	Mean	SD	Mean	SD	Mean	SD
Age (y)	24.76	3.91	24.99	3.23	25.35	4.52	25.30	4.47
FSH (IU/L)	4.73	1.72	5.93	1.42	4.95	3.10	5.53	3.07
LH (IU/L)**	9.19	6.17	9.30	5.09	7.30	8.79	6.20	8.70
LH/FSH**	1.59	0.89	1.26	0.74	1.91	0.72	0.90	0.71
PRL (μg/L)	16.12	54.37	16.80	44.89	13.78	14.29	13.75	14.15
E2 (pmol/L)	198.46	51.36	157.51	42.40	199.58	94.18	220.35	93.23
PRGE (nmol/L)	2.34	1.36	1.85	1.12	1.96	0.88	1.95	0.87
TT (nmol/L)**	2.44	0.41	1.94	0.34	2.33	0.45	1.33	0.45
Insulin (0 h) (μU/mL)**	13.58	6.94	10.77	5.73	15.92	1.46	5.91	1.45
Glucose (0 h) (mmol/L)	4.37	1.29	3.47	1.06	4.71	0.54	4.70	0.53
HOMA-IR**	3.23	1.86	2.05	1.53	3.38	0.36	1.38	0.36

Group I had 32 diabetic women’s with PCOS syndrome (PCOS-D), Group II, includes 38 non diabetic PCOS (PCOS-ND) women, Group III includes 34 Diabetic women but non-PCOS (NPCOS-D). Additionally, 25 age and BMI-matched healthy control (HC) participants were recruited

On analysis of mitochondrial tRNA^Leu^ (UUR) (R = A or G) gene, results indicates ten different type of mutation in study sample, among which only two was seen in HC group and one was seen in non POCS no diabetic group. Most of these mutations (80%) were confined to 3157 to 3275 base region which is evolutionarily conserved region of nucleotides and expected to cause an alteration in the secondary structure of mt-tRNAs (Table 2). As indicated, six mutations (A to G and/or T to C) disrupted putative base pairing. Moreover, we identified two mutations which do not disrupt base pairing occurred in healthy participants but were absent in women with PCOS group suggesting the neutral nature of polymorphisms. The mtDNA copy numbers were considerably lower PCOS group irrespective of diabetic status. Analysis of the copy number PCOS women too had less copy number when compared to the Non-PCOS and/or healthy group (Table 3).

**Table 2:** Details of mutation found in the study sample

Nucleotide position	Nucleotide change	Mutation status	PCOS group		Non-PCOS group	
			Diabetic (n = 32)	Non diabetic (n = 38)	Diabetic (n = 34)	HC (n = 25)
3157	G to A	Homoplasmy	1	1	0	0
3162	C to T	Homoplasmy	0	0	0	0
3203	G to C	Homoplasmy	2	1	0	1
3282	T to C	Homoplasmy	1	1	0	0
3285	T to C	Homoplasmy	1	1	0	0
3302	C to G	Homoplasmy	1	1	0	0
3275	T to C	Homoplasmy	2	2	0	0
4225	A to G	Homoplasmy	1	0	0	0
5206	G to C	Homoplasmy	2	1	0	0
8434	A to T	Homoplasmy	1	0	1	2

**Table 3:** mrDNa copy number comparison

mt copy number			
PCOS group		Non-PCOS group	
Diabetic (n = 32)	Non diabetic (n = 38)	Diabetic (n = 34)	HC (n = 25)
8.78	7.56	9.22	10.1

### Discussion

A number of endocrine abnormalities such as dyspidemia, hyperglycemia and hyperinsulinemia and other such metabolic syndrome occur in PCOS patients [15, 16]. Since insulin resistance and mitochondrial dysfunction has a correlation between each other, it plays a major role in the pathogenesis of PCOS. In glucose metabolism, the mitochondrial function plays a critical role due to which T2DM has the potential pathogenic roles in PCOS. In a number of diseases, the researchers identified point mutations in the functional genes that encode mt-tRNAs [17, 18]. Such tRNA mutations may lead to transcriptional and eventually translational defects which might lead to mitochondrial respiratory dysfunction. In the present study, the researchers sequenced the mtRNA using PCR-Sanger method and identified 10 potential mutations that may occur in PCOS women, especially in the highly conserved regions of the sequence. Thus it is proved that any malfunctions in mt-tRNA may have an involvement in the pathogenesis of PCOS [19]. Still the role played by mitochondrial dysfunction among PCOS patients who carry these mt-tRNA mutations remains unclear [13, 16–19].

From the study results, it can be inferred that PCOS women had significantly high levels of LH, LH/FSH, TT, fasting insulin levels and HOMA-IR when compared to the control group and non-PCOD-D group which denotes their role in PCOS. The mutations were mostly observed in PCOS study sample individuals though they were found in HC group (two mutations) and non-PCOS no diabetic group (one mutation). The most significant result obtained was that 80% of these mutations were confined to core base region of evolutionarily-conserved nucleotides that involve in the development of secondary structure of mt-tRNAs. From the observation, it is understood that mt-tRNA mutations in the pathogenesis of PCOS-IR collapses the secondary structure of mt-tRNA resulting in the failure of mt-tRNA metabolism, impairment of mitochondrial protein synthesis and respiration. The complications discussed above may be the reason behind abnormal mitochondrial respiration which results in the synthesis of low amount of ATPs and eventually pancreatic β-cell dysfunction and apoptosis finally resulting in the reduced amount of insulin secretion.

In PCOS group, the mtDNA copy numbers were comparatively less irrespective of whether diabetic or not. But on the contrary, some researchers place their argument that mtDNA mutation is a variant of homoplasmic and have no role in the abnormalities of PCOS or diabetes and association with MIDs. Finsterer et al. [20] reported that when mtDNA gets depleted, it may cause PCOS. This claim was heavily supported i.e., when mtDNA gets depleted, it is generally associated with a number of clinical manifestations such as CPEO, Pearson syndrome, Kearns-Sayre syndrome. During the age of reproduction, women face infertility issues in the presence of PCOS and are attributed to their endocrine disorders. Those females might be exposed to high risk of developing cardiovascular disease or diabetes [21, 22]. Diabetes follows every generation and has an association with PCOS. Women with PCOS are resistant to insulin and predicted to have diabetes in their latter ages. A number of studies tracked the history of diabetes among PCOS families. The studies conducted recently showed a correlation between mitochondrial leucine tRNA gene mutation and diabetes.

According to the researchers [23–25], when there is damage occurs in mtDNA, it can be a measure to identify mitochondrial dysfunction since the former leads to reduced cellular metabolic activity. In the recent times, studies proved the mtDNA mutations, its effect on mitochondrial dysfunction and the role played in the pathogenesis of PCOS-IR [13]. Mitochondrial Disorders predominantly occur due to the gene mutations in mitochondrial DNA and/or the nuclear DNA (nDNA). MIDs are usually heterogeneous in nature and highly influence the metabolism, maintenance or signaling of mitochondrian proteins [26–28].

### Conclusion

The current study was aimed at analyzing the mtDNA mutations and their copy number in order to measure the impact created by Diabetes among PCOS women. There is an association between PCOS and a number of genetic disorders and the prevalence is high with MIDs (mitochondrial disorders).

## Limitation

The important limitation of this study is that the study has been conducted with a relatively small population. So, the studies to be conducted in future must have a large sample size so as to establish strong evidence between MIDs and PCOS. Further, PCOS was not reported earlier in a patient with specific or non-specific. The evidence obtained from the study cannot be confirmed that PCOS is a phenotypic feature of MIDs. The current study conducted on mtDNA variants reported a small sample size associated with PCOS using a single centre can be justified with large sample size in multicenter studies as well.

## Data Availability

All associated data are available in this manuscript and no data in placed other than what is provided.

## Abbreviations

POC: polycystic ovary syndrome
ROS: reactive oxygen species
BMI: Body Mass Index
WHR: waist by hip ratio
FSH: follicle stimulating hormone
LH: Luteinizing hormone
E2: estradiol
mt-tRNA genes: MT-TL1 mitochondrially encoded tRNA leucine
